# Functional Analysis of *VDR* Gene Mutation R343H in A Child with Vitamin D-Resistant Rickets with Alopecia

**DOI:** 10.1038/s41598-017-15692-z

**Published:** 2017-11-10

**Authors:** Min-Hua Tseng, Shih-Ming Huang, Fu-Sung Lo, Jing-Long Huang, Chih-Jen Cheng, Hwei-Jen Lee, Shih-Hua Lin

**Affiliations:** 1 Graduate Institute of Medical Sciences, National Defense Medical Center, Taipei, Taiwan; 2 Division of Pediatric Nephrology, Department of Pediatrics, Chang Gung Memorial Hospital-Chang Gung University, Taoyuan, Taiwan; 3 Department of Biochemistry, National Defense Medical Center, Taipei, Taiwan; 4 Division of Pediatric Endocrinology, Department of Pediatrics, Chang Gung Memorial Hospital-Chang Gung University, Taoyuan, Taiwan; 5 Division of Allergy, Asthma and Rheumatology, Department of Pediatrics, Chang Gung Memorial Hospital-Chang Gung University, Taoyuan, Taiwan; 6 Division of Nephrology, Department of Medicine, Tri-Service General Hospital, National Defense Medical Center, Taipei, Taiwan

## Abstract

The functional study of different mutations on *vitamin D receptor (VDR)* gene causing hereditary vitamin D-resistant rickets (HVDRR) remains limited. This study was to determine the *VDR* mutation and the mechanisms of this mutation-causing phenotype in a family with HVDRR and alopecia. Phenotype was analyzed, and *in vitro* functional studies were performed. The proband and his affected sister exhibited typical HVDRR with alopecia, and their biochemical and radiographic abnormalities but not alopecia responded to supraphysiological doses of active vitamin D_3_. A novel homozygous missense R343H mutation in the exon 9 of *VDR* residing in the retinoid X receptor (RXR)-binding domain was identified. The expression level and C-terminal conformation of R343H mutant are not different from the wild-type VDR. This mutant had no effect on the nuclear localization of VDR, VDR-RXR heterodimerization, but it impaired *CYP24A1* promoter activity in the presence of 1,25 (OH)_2_ vitamin D_3_, at least in part, mediated through specific nuclear receptor coactivator. Simulation models revealed the vanished interaction between guanidinium group of R343 and carboxyl group of E269. Without affecting the expression, conformation, nuclear location of VDR or heteridimerization with RXR, VDR-R343H impairs the transactivation activity of VDR on downstream transcription, accounting for HVDRR features with alopecia.

## Introduction

Hereditary vitamin D-resistant rickets (HVDRR) is an autosomal recessive disease characterized by hypocalcemia, hypophosphatemia, secondary hyperparathyroidism, and an increased serum 1,25-dihydroxyvitamin D3 (1,25(OH)_2_D_3_; Vit D3) level^1–3^. Patients with HVDRR usually manifest rickets during early infancy and progressively develop bone pain, muscle weakness, short stature, and occasional alopecia^2^. Loss of function mutation in the *vitamin D receptor* (*VDR*) gene that encodes VDR is well known to cause HVDRR. VDR, as the mediator of action of Vit D3, is composed of DNA-binding and ligand-binding domains for Vit D3, retinoid X receptor (RXR), and coactivators^4^. More than 50 different *VDR* mutations, including missense, nonsense, deletion, and splicing mutations, in all binding domains of VDR have been identified. Most of these mutations are missense and are primarily located at the ligand-binding domain of VDR. Patients with mutations in different binding domains have been shown to exhibit distinct and variable severity of phenotypes^5,6^. The study of the VDR mutant on RXR-binding domains remains limited.

We recently encountered a family with clinical diagnosis of HVDRR featuring early-onset rickets and alopecia accompanied by hypocalcemia, hypophosphatemia, secondary hyperparathyroidism, and extraordinarily high levels of Vit D3. In this study, we performed genetic analysis of the *VDR* gene in this family and a functional study was performed to explore the molecular mechanism *in vitro* to aid in understanding the pathogenesis of HVDRR.

## Results

### Phenotype of patient

**Figure 1:**
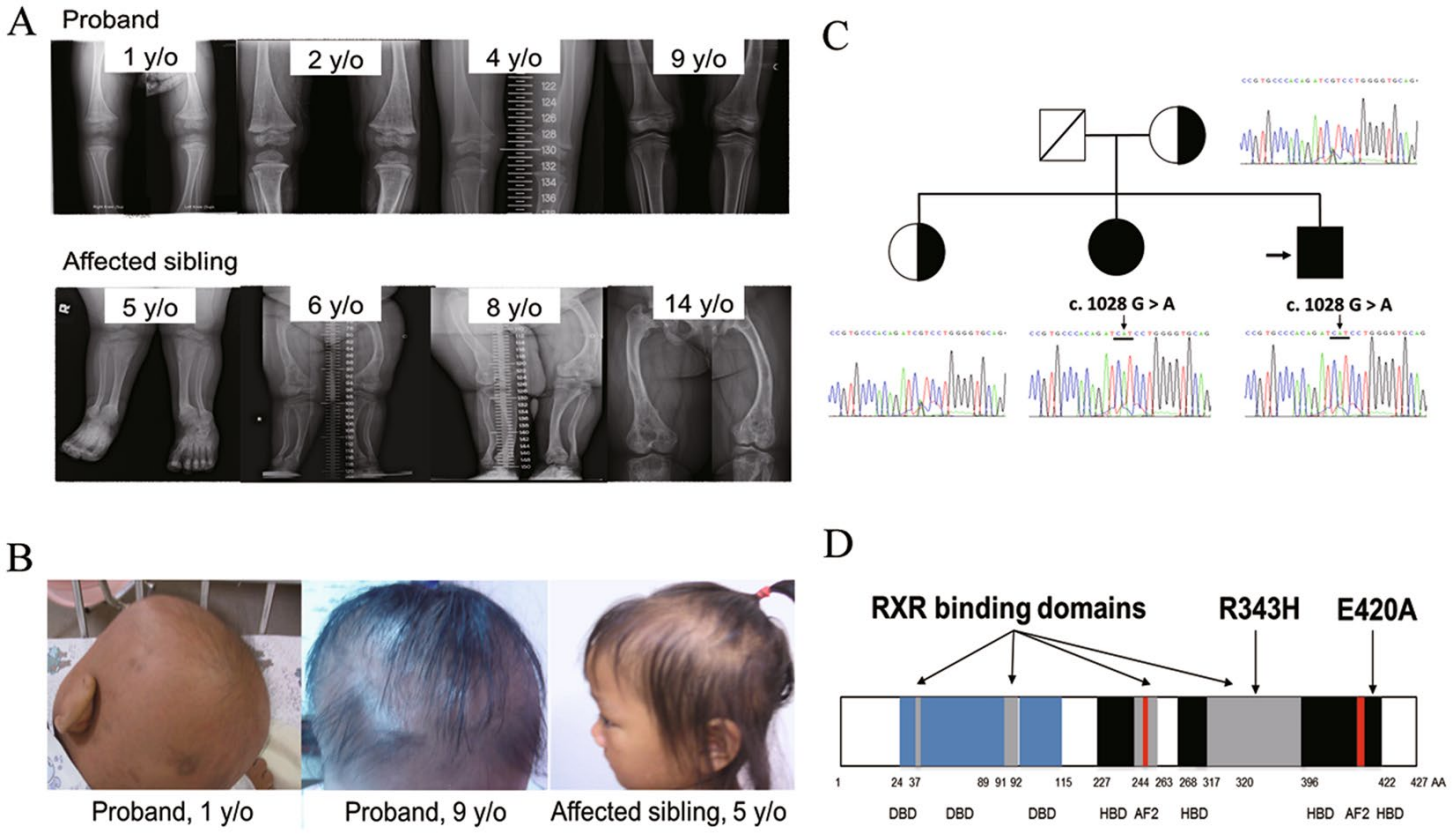
Summary of clinical features, genetic analysis of *VDR*, and functional domains of VDR. (**A**) Rickets. Sequential bone radiographs of proband (upper row) and affected sibling (low row). (**B**) Alopecia. (**C**) Direct sequencing for *VDR* gene. Filled symbols and arrow represent affected individuals and homozygous mutant base, respectively. (**D**) Mutated amino acid and functional domains of VDR. Blue, black, and red symbols indicate DNA binding, hormone binding, and AF2 binding domains, respectively.

The proband, shown in Fig. 1 at 9 years of age, was born to a healthy 30-year-old mother with no family history of consanguinity or rickets. He had an uneventful full-term delivery with normal birth weight and height and was fed with standard formula milk after birth. From the age of 1 month, sparse hair with progressive loss was noted on the scalp. At 1 year of age, bowed legs developed, and a radiograph of the knees was consistent with rickets. Hypocalcemia and hypophosphatemia with a remarkably increased serum level of Vit D3 and intact parathyroid hormone and alkaline phosphatase levels confirmed the diagnosis of HVDRR (Table 1). A combination of oral calcium, phosphate, and calcitriol was given and adjusted based on the biochemical and radiographic responses. The dosages of calcium, phosphorus, and calcitriol were increased to 200 mg/kg/day, 100 mg/kg/day, and 15 μg/day, respectively. His serum calcium and phosphate levels increased and his serum intact parathyroid hormone level had begun to decline gradually at 4 years of age. Biochemical studies and sequential radiographs obtained at follow-up are shown in Table 1 and Fig. 1, respectively. His short stature had improved at 9 years of age, but the alopecia persisted.

**Table 1:** Sequential laboratory and therapeutic responses in proband and affected sister.

Serum	Normal range	Proband		Affected sister	
		1 year^*^	9 years	5 years^*^	13 years
Creatinine	0.25–0.90 mg/dL	0.20	0.44	0.3	0.39
Total Calcium	8.5–10.5 mg/dL	6.4	7.9	6.4	8.2
Phosphate	4.0–6.0 mg/dL	3.2	6.1	3.3	3.4
Magnesium	1.3–2.1 mg/dL	1.6	1.8	1.6	1.7
Alkaline-P	149–435 U/L	1047	604	2179	772
Albumin	2.8–4.0 g/dL	3.2	4.9	3.6	4.7
25 OH Vit D_3_	9–42 ng/mL	20.3	5.2	24.4	6.9
1,25 (OH)_2_ Vit D_3_	15–45 pg/mL	170	>200	184	>200
Intact PTH	12–72 pg/mL	308	92	442	64
Treatment					
Calcium	mg/kg/day	100	75	90	60
Phosphate	mg/kg/day	75	—	75	—
Calcitriol	μg/day	6	12	5	10

*Before treatment.

The proband’s affected sister exhibited similar phenotypes, including early-onset alopecia, rickets, and follow-up results (Table 1 and Fig. 1). The patients’ parents and one sister are phenotypically unaffected.

### *VDR* gene mutation

Direct sequencing of the genomic DNA revealed a single base substitution at position 1028 (c. 1028 G > A) in exon 9, resulting in amino acid substitution of arginine for histidine (R343H) in the proband and his affected sister (Fig. 1C). This point mutation was located on the RXR binding domains of VDR (Fig. 1D). This variant, *VDR* (ENST00000229022.7): c.1028 G > A (R343H), was absent in 200 healthy Taiwanese.

### Analyses of the VDR expression

**Figure 2:**
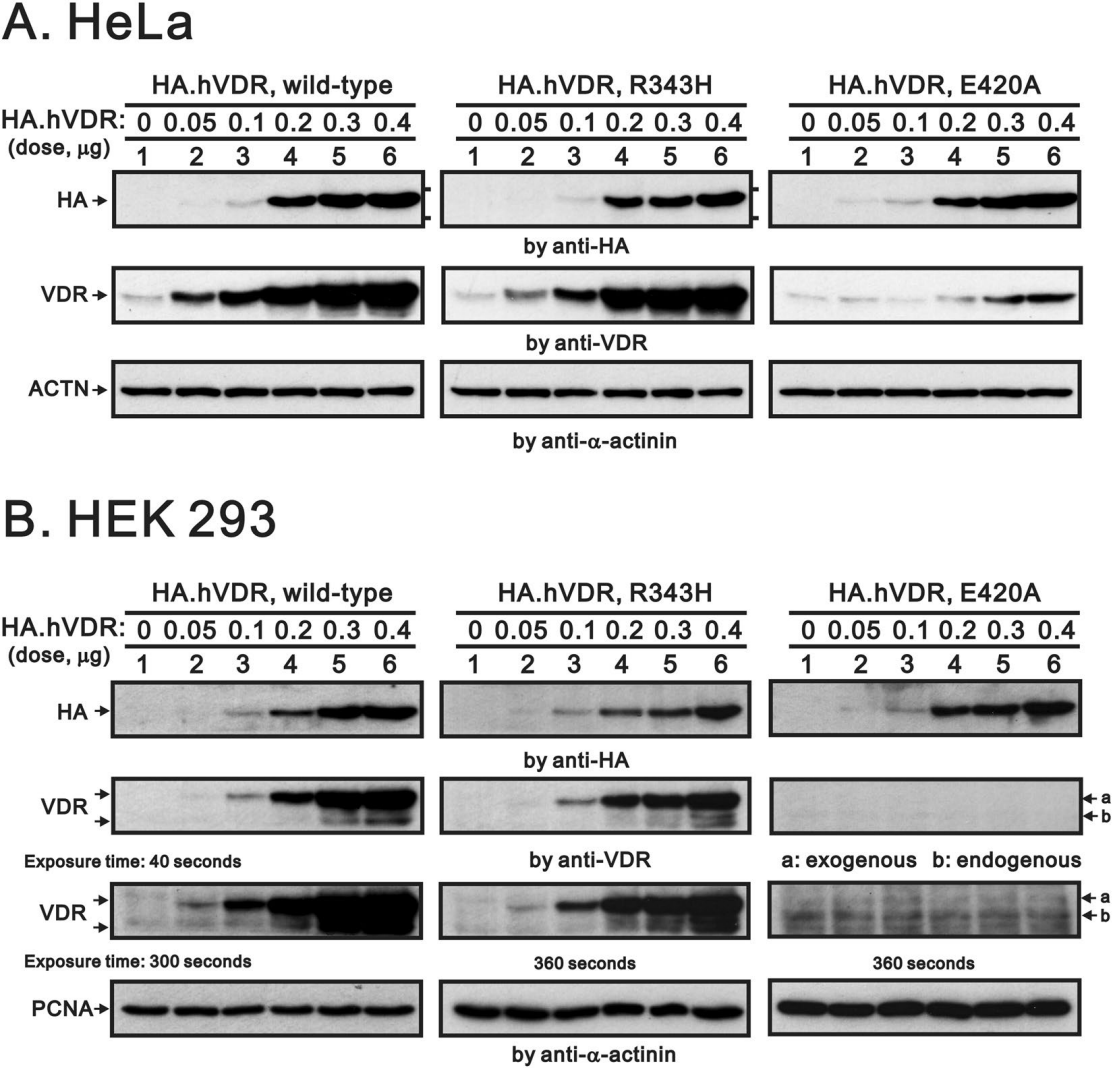
Expression of VDR. Both HeLa (**A**) and HEK293 (**B**) cells were transfected with pSG5.HA.VDR–wild type, pSG5.HA.VDR–R343H and pSG5.HA.VDR–E420A expression constructs. Cell extracts were subjected to Western blot analysis probing with antibodies against anti-HA and the amino acid residues 343–424 of VDR. Antibodies to α-actinin and proliferating cell nuclear antigen were for loading control for HeLa and HEK293 cells, respectively. Various indicated exposure time scales were for the VDR antibody analysis in HEK 293 cells. Results are representative of three independent experiments.

The expressions of VDR–wild type, VDR–R343H, and VDR–E420A proteins were enhanced by overexpressed VDR in a dose-dependent manner via analysis of the anti-HA antibody in both HeLa and HEK293 cell lines (Fig. 2). The expression of HA-tagged VDR–R343H and HA-tagged VDR–E420A proteins was relatively equal in band density compared with VDR–wild type, although the detectable amounts of VDR–E420A proteins were far less than VDR–wild type and VDR–R343H proteins, using an antibody against the C-terminal region of VDR in both HeLa and HEK293 cell lines. These results indicate that the expression level and C-terminal conformation of R343H mutant are not different from those of wild-type VDR.

### VDR expression in the presence of 1,25 (OH)_2_ Vitamin D_3_

**Figure 3:**
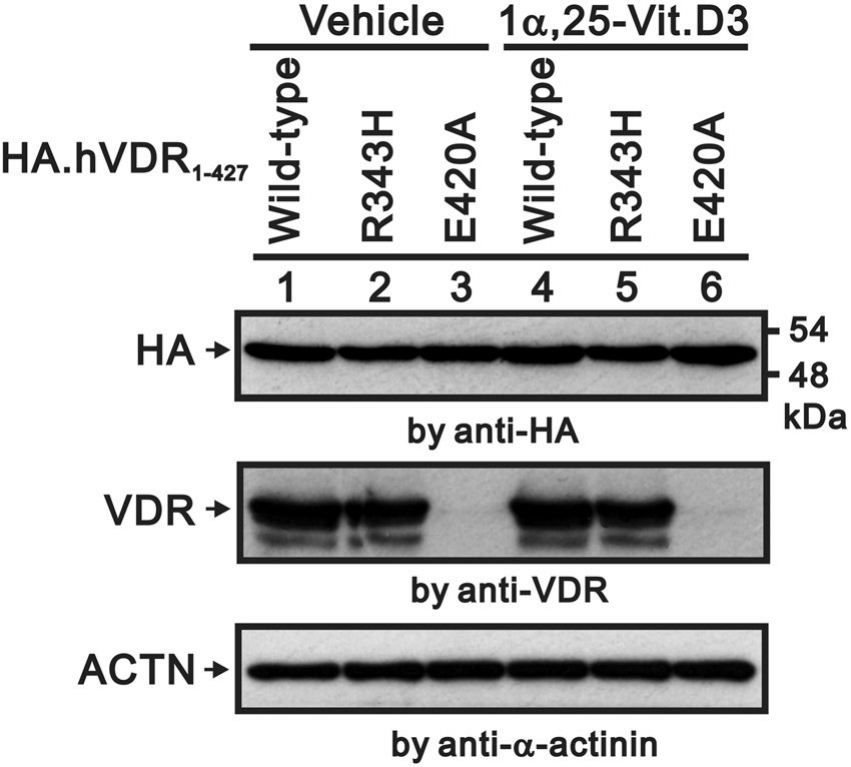
Expression of VDR in presence and absence of 1,25(OH)_2_D_3_. HEK 293 cells were transfected pSG5.HA.VDR–wild type, pSG5.HA.VDR–R343H, and pSG5.HA.VDR–E420A expression constructs. Cells were treated with vehicle or Vit D3. Cell extracts subjected to Western blot analysis were probed with respective antibodies to anti-HA antibody and antibody against amino acid residues 343–424 of VDR. Results are representative of two independent experiments.

The expression levels of VDR–wild type, VDR–R343H, and VDR–E420A proteins did not differ in amount in the presence or absence of Vit D3 stimulation (Fig. 3). This finding indicates that Vit D3 did not affect the expression of VDR.

### VDR localization

**Figure 4:**
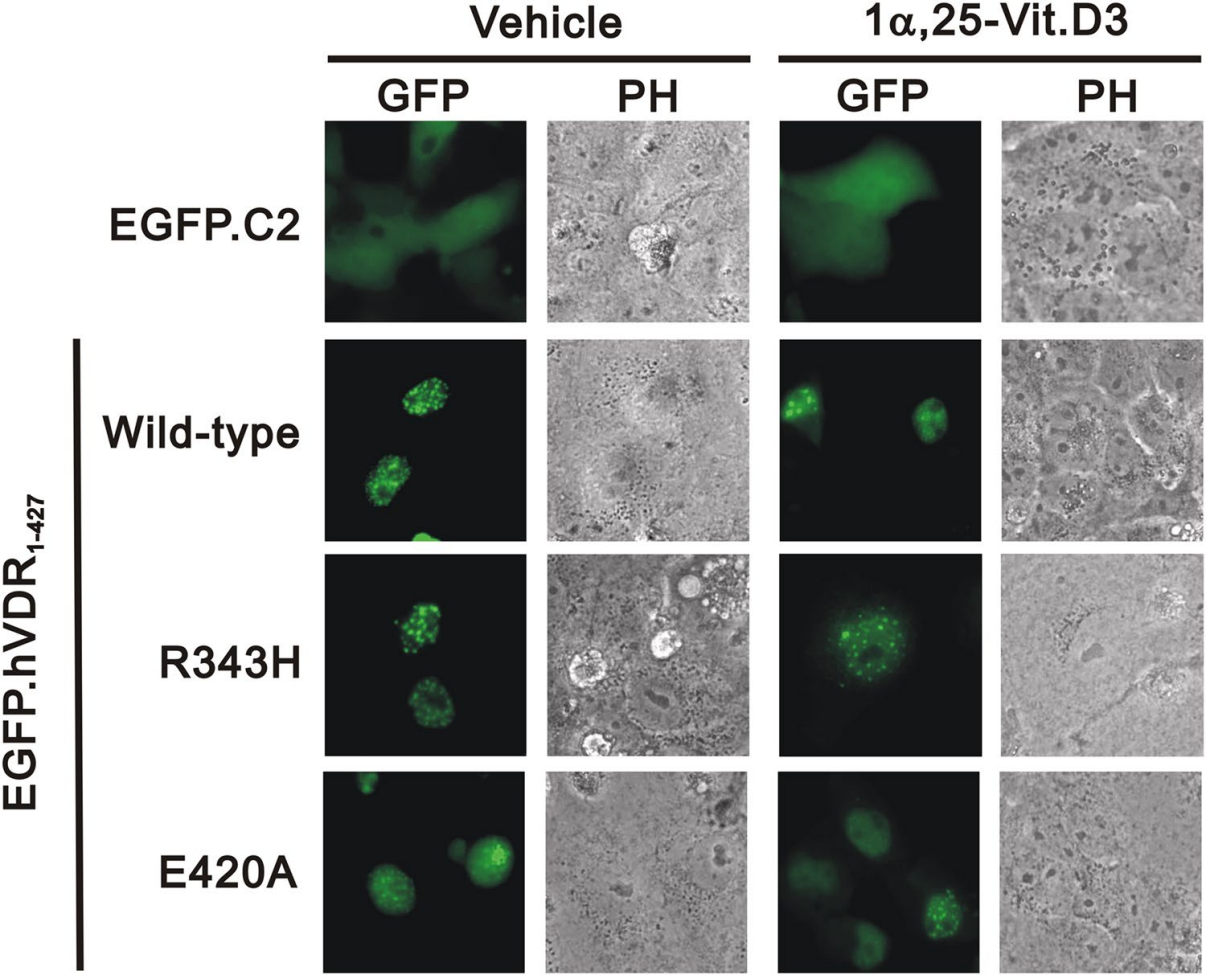
Nuclear localization of mutant VDR. HeLa cells were transiently transfected with pEGFP vector or pEGFP.VDR–wild type, pEGFP.VDR–R343H, and pEGFP.VDR–E420A. Approximately 100 cells were examined, and each nuclear localization of EGFP fusion proteins was recorded under florescence microscopy. Results are representative of three independent experiments.

Because VDR itself is a transcriptional factor, the mutant VDR–R343H was evaluated as to whether it impaired nuclear localization in cells. We generated EGFP fusion proteins and determined the subcellular location of various VDR proteins by florescence microscopy. As shown in Fig. 4, neither R343H nor E420A disrupted the nuclear localization, which suggests that the phenotype in this proband did not result from the impairment of nuclear localization.

### VDR transactivation activity

**Figure 5:**
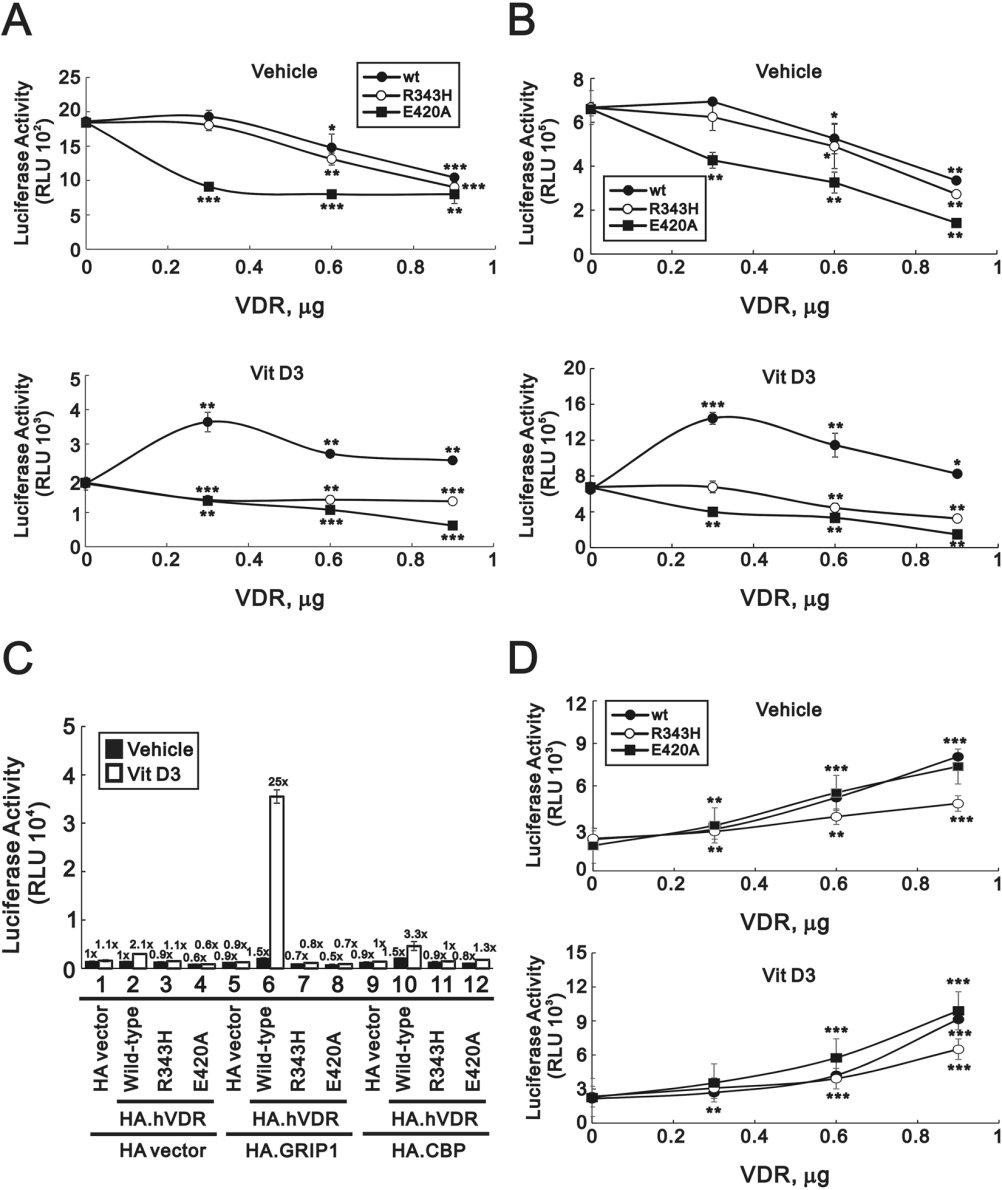
Transactivation of VDR. (**A**) HeLa and (**B**) HEK293 cells were co-transfected with *CYP24A1*-luciferase reporter gene (0.2 μg) and indicated amount of pSG5.HA.VDR–wild type, pSG5.HA.VDR–R343H, and pSG5.HA.VDR–E420A constructs. (**C**) HeLa cells were co-transfected with *CYP24A1*-luciferase reporter gene (0.25 μg) and 0.25 μg of pSG5.HA, pSG5.HA.VDR–wild type, pSG5.HA.VDR–R343H, or pSG5.HA.VDR–E420A in the presence of pSG5.HA, pSG5.HA.GRIP1, pSG5.HA.CBP. (**D**) HeLa cells were co-transfected with *p21*-luciferase reporter gene (0.2 μg) and indicated amount of pSG5.HA.VDR–wild type, pSG5.HA.VDR–R343H, and pSG5.HA.VDR–E420A constructs. Cells were treated with vehicle or 0.1 µM Vit D3 for 20 h and harvested for luciferase reporter assays. Data are average of three experiments (mean ± SD; n = 3). The numbers above the (**C**) columns indicate the luciferase activity relative to an index of 1 for the pSG5.HA vector alone with vehicle.

To study whether this VDR–R343H mutant could affect the transactivation activity of the wild type of VDR, we generated a luciferase-reported assay driven by the native promoter of human *CYP24A1*, one of the verified VDR-regulated genes in human cells, as the surrogate gene expressed in cells. Our results show that the transactivation of VDR could be evaluated by determining the *CYP24A1* promoter activity in the presence and absence of Vit D3. As shown in Fig. 5A and B, VDR–wild type, VDR–R343H, and VDR–E420A proteins could differentially repress *CYP24A1* promoter activity in the absence of Vit D3, whereas the Vit D3–activated VDR–wild type protein, but not the VDR–R343H or VDR–E420A proteins, could enhance *CYP24A1* promoter activity in HeLa and HEK293 cells.

We further examined whether VDR–R343H as well as VDR–E420A had disrupted protein-protein interaction with nuclear receptor coactivators, such as glucocorticoid-receptor-interacting protein (GRIP1) and cAMP-response-element-binding protein (CBP). Our results showed that only Vit D3-activated VDR–wild type synergistically enhanced the *CYP24A1* promoter activity with GRIP1 in HeLa cells (Fig. 5C, compare with histograms 1 and 6). Our results suggest that both VDR–R343H and VDR–E420A might lose the ability to recruit some nuclear receptor coactivators for the transactivation activity of VDR. We also checked another VDR target gene, *p21*, and found that VDR–E420A, not VDR–R343H, worked well as VDR–wild type on the *p21* promoter activity in the absence and presence of Vit D3 (Fig. 5D). Summarily, our findings demonstrate that the VDR–R343H mutant protein might have different functions via alternative regulation of the VDR target genes.

### VDR and RXR heterodimerization

**Figure 6:**
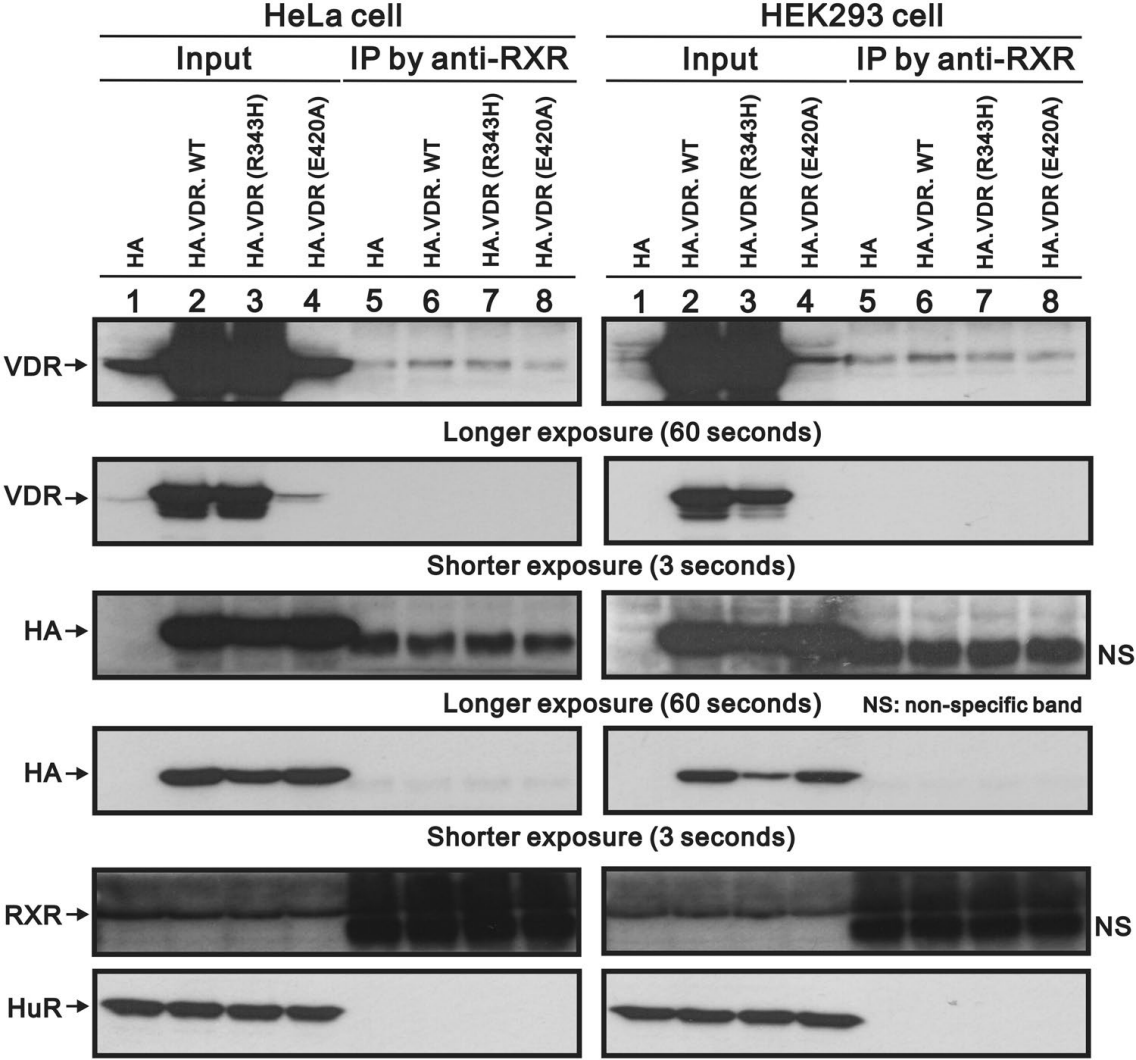
Physical interaction between various VDR and RXR. HeLa and HEK293 cells were transfected with SG5.HA.VDR-wild type, pSG5.HA.VDR-R343H, and pSG5.HA. Cells harvested after the transfection were immunoprecipitated with RXR antibody complexed with protein A/G magnetic beads, washed, eluted and processed by western blotting with antibodies against HA or VDR. HuR was a negative control and RXR was a positive control.

Because of the importance of the heterodimerization of VDR and RXR for VDR transactivation and the mutation on RXR-binding domain, we further analyzed the possible interaction defect between RXR and VDR–R343H by using co-immunoprecipitation assay (Co-IP). As shown in Fig. 6, no difference in heterodimerization with RXR between VDR–R343H and VDR–wild type in the absence or presence of Vit D3. This result indicated that this R343H might not abolish the VDR-RXR heterodimerization.

### Simulation models of RXR-VDR LBD heterodimer

**Figure 7:**
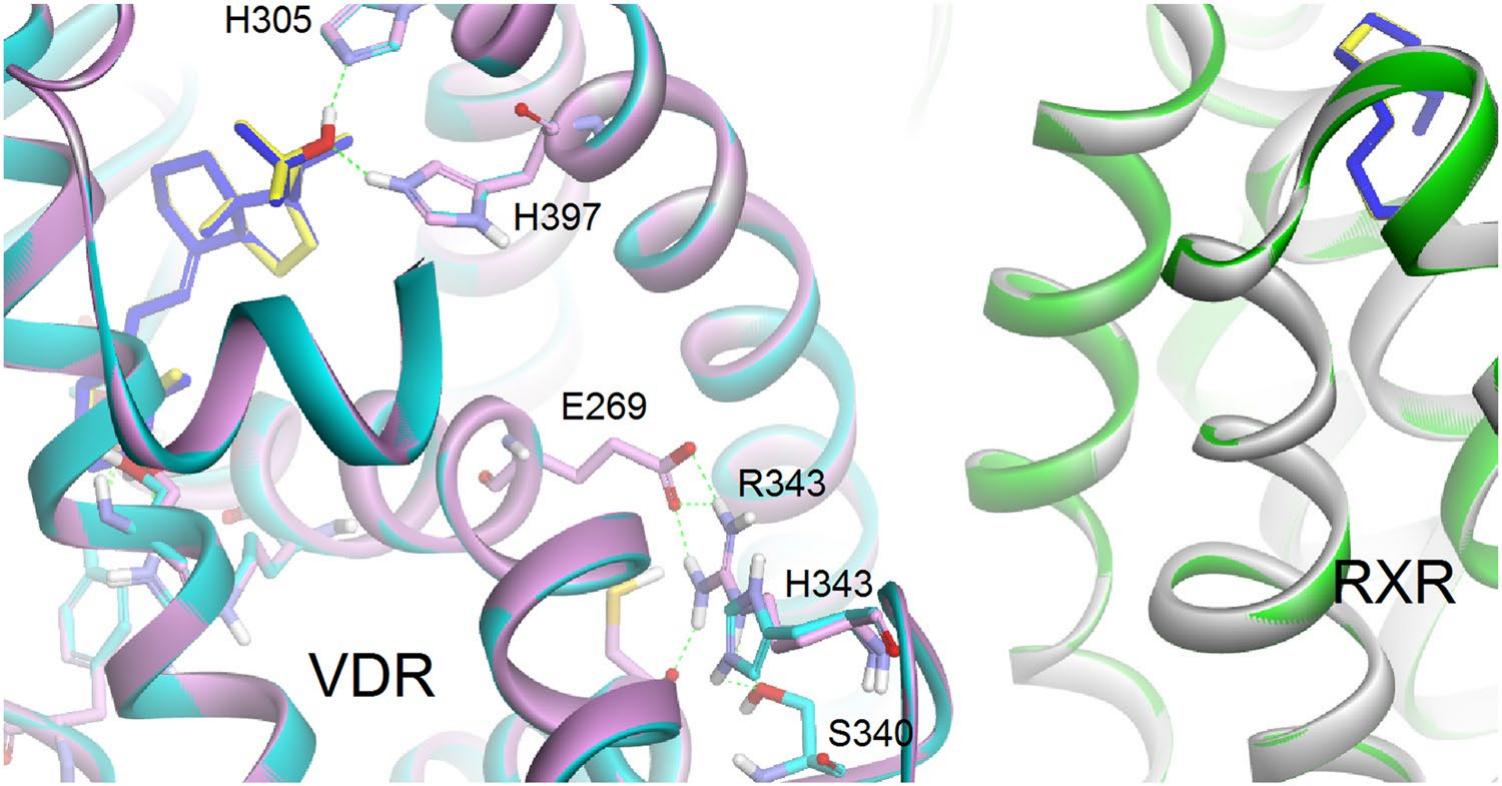
Simulation models of RXR-VDR LBD heterodimer. Superimposition of wild-type with R343H mutant VDR protein. Wild-type and mutant proteins are shown as ribbon model and are colored white and pink for RXR and green and cyan for VDR. Ligands are represented by stick model and are colored yellow for wild type and blue for mutant protein. Hydrogen bond interactions are highlighted as dotted green lines.

To elucidate the possible mechanisms of abolished VDR transactivation caused by R343H, we analyzed the simulation models. The residues of Y143, S237, R274, S278, H305, and H397 in the binding site of VDR interacted with Vit D3. The R343 was located in the interhelical loops between H8 and H9. The guanidinium group of R343 forms a hydrogen bond with the carboxyl group of E269, which is located in helix 5 of VDR (Fig. 7). The interaction vanished for the R343H mutant.

## Discussion

We identified a novel homozygous mutation R343H on the *VDR* gene in a family with typical HVDRR and alopecia. This amino acid 343 arginine residue (R343) on VDR should play an important role in calcium homeostasis, bone mineralization, and normal hair development. In an *in vitro* study, this mutation located on the RXR-binding domain of VDR expressed well and had similar C-terminal conformation as wild-type VDR. It also did not alter the nuclear localization, and VDR-RXR heterodimerization, but did impair the Vit D3–dependent transactivation of the downstream target gene, *CYP24A1*, via specific nuclear receptors.

The functional study of the reported VDR mutants reveals the molecular mechanisms in HVDRR^7^. Mutations in the DNA binding domain prevent the interaction of VDR and vitamin D response element (*VDRE*) of target genes^3^, and mutations in the vitamin D_3_ and transactivation binding domains affect the binding of hormone and coactivators, respectively^8,9^. At least four RXR-binding sites are located in VDR, including 37, 91–92, 244–263, and 317–395 binding region (Fig. 1D)^10^. Mutations in RXR-binding domains may reduce the expression of VDR or cause a defect in the heterodimerization of VDR and RXR^11–13^. Although this R343H mutant had been reported in case of heritable rickets by Rauch *et al*.^14^, the functional assay of this mutant is lack. This identified VDR-R343H mutation in the RXR-binding domain did not affect the expression level of VDR protein as comparing with wild-type VDR. The distinct mutation sites in the RXR-binding domain have shown a different degree of attenuation in VDR-RXR heterodimerization, resulting in variable turnover of mutant VDR. We used E420A mutant, another ligand-binding domain of VDR, due to its normal affinity with RXR^15^. Unlike E420A, this R343H mutation did not alter the conformation of the transactivation-binding domain of VDR. Neither E420A nor R343H altered the nuclear localization of VDR. Accordingly, the R343H mutant did not reduce VDR expression, change the conformation of its C-terminal, or impair its nuclear translocation.

To study whether Vit D3–dependent transactivation was involved in R343H to cause HVDRR, we evaluated *CYP24A1* promoter activity as one of the well-known VDR-regulated genes in human cells. The VDR–R343H like E420A mutant impaired Vit D3–dependent transactivation. The abolishment of the transactivation activity of VDR–E420A mutant has been shown by defective interactions of VDR with coactivators including SRC-1 and DRIP205^16,17^. In this mechanism, which is similar to that of VDR–E420A and other VDR-RXR binding domain mutants, VDR–R343H should reasonably impede the transactivation activity^11,12,18,19^. Deletion studies of VDR have demonstrated that the formation of heterodimer with RXR is necessary for transcriptional activity^20–22^. From crystallographic studies, the dimer interface of VDR-RXR is formed from helixes 9 (H9) and 10 (H10) and the interhelical loops between H7 and H8 and H8 and H9 of VDR^23^. Because VDR-R343 lies in the interhelical loop between H8 and H9, this critical mutation site would impair the heterodimer interface and disturb heterodimerization. However, we demonstrated that this VDR-R343H did not disrupt the VDR-RXR heterodimerization by using three different Co-IP experimental conditions, including overexpression of RXR. The condition of exogenously overexpressing RXR and various VDRs seem to rule out the endogenous RXR contributed to most of the VDR pull-down by RXR antibody in our case. This blunt result may be the truth for current status. The in *vitro* GST pull-down or proximity ligation assays may overcome the limitation of traditional Co-IP experiment. Simulation models revealed that the alteration of charge interaction between R343 and E269 resulted in instability of the H5 structure of VDR, which is important for the constitution of hydrophobic cleft with H3 and H4, by this novel mutation. This cleft has been demonstrated to hold the competitive binding sites of transcriptional coactivator and co-repressor molecules^24^. This was supported by our findings that the synergistic enhancement on the *CYP24A1* promoter activity when the recruitment of transcriptional co-activator, GRIP1, by ligand-activated VDR–wild type, not VDR-R343H mutant.

The early-onset alopecia also found in the proband and affected sibling with HVDRR, which was consistent with case of mutation R343C reported by Mazen *et al*., may be associated with the severity of the vitamin D resistance^25^. Alopecia can be variable (totalis or partialis) in appearance and extent, and its exact pathogenesis in HVDRR remains to be elucidated. Mice and patients who express a truncated VDR that lacks a DNA-binding domain exhibit alopecia, and patients with mutations in RXR but not in the Vit D3 ligand–binding domain also have alopecia^26–28^. Recently, the hair follicle cycle was reported to be also regulated by unliganded VDR, and defects in the interaction between unliganded VDR and the HR, Lef1, β-catenin, Hh, and Wnt signaling pathways impair the hair cycle^9,29–33^. The alopecia in patients with HVDRR, including our patient, was persistent and did not respond to active vitamin D_3_ therapy^5,6^. Further therapies that target the enhancement of interaction between guanidinium group of R343 and carboxyl group of E269 by either genetic therapy with correction of this point mutation of VDR might prove to be a curative treatment for alopecia in this family with R343H mutation of *VDR* ^34–36^.

With respect to therapy and follow-up in this family with HVDRR, normalization of biochemical features and improvement of rickets were observed after supraphysiological doses of oral calcitriol and calcium treatment, as mentioned in previous reports of patients with RXR-binding domain mutations^17,19,20^. The responsiveness of Vit D3 supplementation in patients with HVDRR appears to depend on the mutation sites of *VDR*. The VDR function in patients with Vit D3 and RXR-binding domain mutations could be rescued by ultra-physiological doses of calcitriol, whereas those with DNA-binding domain mutations have complete hormone resistance even with extraordinarily high doses of calcitriol^1,37,38^. Of interest, our patient did not require oral phosphorus supplementation following the correction hypocalcemia and secondary hyperparathyroidism, indicating that hypophosphatemia in patients with HVDRR is highly related to vitamin D resistance and secondary hyperparathyroidism per se^5,6^.

## Conclusion

We identified a novel homozygous R343H mutation in the RXR-binding domain of *VDR* in a family with HVDRR and refractory alopecia. This VDR–R343H mutation resulted in abolishment of Vit D3–dependent transactivation, which was not related to the defect of VDR-RXR heterodimerization but result from the failure of transcriptional co-factors recruitment that may be caused by instability of the H5 structure of VDR. This study provides additional insight into the effects of VDR structure defects on the functional consequences of calcium homeostasis, bone mineralization, and hair follicle development.

## Methods

### Patient

This study followed the tenets of the Declaration of Helsinki, and was approved by the ethics committee on human studies at Chang Gung Memorial Hospital in Taiwan (IRB105-6067C). All methods were performed in accordance with approved guidelines. Written informed consent including publication of identifying image in an open-access journal was obtained from the guardian of children involved after a detailed description of the study.

### Direct sequencing of *VDR* gene

Genomic DNA was extracted from peripheral leukocyte in this patient and family members with a DNA isolation kit (QIAamp Blood Kit; Qiagen, Dusseldorf, Germany). Exons 2–9 of the *VDR* gene were amplified by polymerase chain reaction with primers and directly sequenced.

### Construction of plasmids

Various *VDR* coding regions were synthesized by polymerase chain reaction and subcloned into the pSG5.HA vector with *EcoR*I and *Xho*I sites or enhanced green fluorescent protein (pEGFP) with *EcoR*I and *Sal*I sites (Clontech Laboratories, Mountain View, CA). The E420A, the other ligand-binding domain, and wild-type VDR were synthesized as negative and positive controls, respectively. The native promoter of the *CYP24A1* gene (−350/−1) used to drive the expression of the construct-carrying luciferase reporter gene was synthesized by polymerase chain reaction and subcloned into the *Xho*I and *Hind*III sites of the pGL3 basic-Luc vector (Promega, Madison, WI). Expression vectors for GRIP1 and CBP in the pSG5.HA vector and *p21*-luciferase reporter gene had been described previously^39,40^.

### Cell culture, transfection and reporter assay

The HeLa and HEK293 cells were grown in Dulbecco’s modified Eagle’s medium supplemented with 10% charcoal/dextran-treated fetal bovine serum. The cells were transfected into 24-well plates with jetPEI (Polyplus Transfection Inc., New York, NY) according to the manufacturer’s protocol. The total DNA was adjusted to 1.0 μg by the addition of the empty vector. The cells were co-transfected with *CYP24A1*- or *p21*-luciferase reporter gene (0.2 μg) and 0.3 μg pSG5.HA.VDR–wild type, pSG5.HA.VDR–R343H, and pSG5.HA.VDR–E420A constructs. The cells were treated with vehicle or 0.1 µM Vit D3 for 20 h and were harvested for luciferase reporter assay, which was performed with the Promega Luciferase Assay Kit; the values are expressed numerically as relative light units. The luciferase activity is presented as the mean ± SD of three transfected wells and is representative of at least three independent experiments.

### Immunofluorescence microscopy

The COS7 cells were grown in Dulbecco’s modified Eagle’s medium supplemented with 10% charcoal/dextran-treated fetal bovine serum. The cells were transiently transfected with 0.3 μg pEGFP vector or pEGFP.VDR–wild type, pEGFP.VDR–R343H, and pEGFP.VDR–E420A. The cells were treated with vehicle or 0.1 µM Vit D3 for 20 h. Approximately 100 cells were examined, and each nuclear localization of EGFP fusion proteins was recorded with a florescence microscope (Model DMIRE2, Leica, Wetzlar, Germany) and analyzed with Image-Pro Plus software (Media Cybernetics, Rockville, MD) 24 h after transfection. The results are representative of two independent experiments.

### Western blotting

The cell lysates were prepared in a lysis buffer (100 nmL^−1^ Tris-HCl, pH 8.0; 150 mmolL^−1^ NaCl; 0.1% sodium dodecyl sulfate; and 1% Triton X−100) at 4 °C, separated by sodium dodecyl sulfate polyacrylamide gel electrophoresis, and transfected onto a polyvinylidene difluoride membrane (Millipore, Bedford, MA). The following proteins were detected using specific antibodies: hemaglutinin (HA) epitope (3F10; F. Hoffman-La Roche, Basel, Switzerland), proliferating cell nuclear antigen, α-actinin, and VDR (F-2, H-2, and D-6; Santa Cruz Biotechnology, Santa Cruz, CA). For evaluation of VDR expression, the HeLa and HEK293 cells were transfected with 0, 0.05, 0.1, 0.2, 0.3, and 0.4 μg of transfected pSG5.HA.VDR–wild type, pSG5.HA.VDR–R343H, and pSG5.HA.VDR–E420A expression constructs for 16 h. The cell extracts were subjected to Western blot analysis probing with antibodies to α-actinin; proliferating cell nuclear antigen; anti-HA antibodies for HA-tagged VDR–wild type, HA-tagged VDR–R343H, and HA-tagged VDR–E420A; and antibodies against the amino acid residues 343 to 424 of VDR. For analysis of VDR expression in the presence of Vit D3, the HEK 293 cells were transiently transfected with 0.3 μg pSG5.HA.VDR–wild type, pSG5.HA.VDR–R343H, and pSG5.HA.VDR–E420A expression constructs. The cells were treated with vehicle or with 0.1 μM Vit D3 for 20 h. The cell extracts were subjected to Western blot analysis probing with antibody to α-actinin and anti-HA antibodies for the HA-tagged VDR–wild type, HA-tagged VDR–R343H, and HA-tagged VDR–E420A. The results are representative of three independent experiments.

### Co-immunoprecipitation assay

The Co-immunoprecipitation assay (Co-IP) assay was performed using Pierce^TM^ Crosslink Magnetic IP/Co-IP Kit (Waltham, MA), according to the manufacturer’s instructions. In brief, HeLa and HEK293 cells were co-transfected with vectors expressing VDR (wild-type, R343H and E420A mutants; 4 μg). Cells were harvested 24 hours after the transfection, washing cells one time by PBS, cells were harvested in ice-cold IP Lysis/Wash Buffer (containing protease inhibitor cocktail). Total cell lysates were collected by centrifugation at 13,000 g for 10 minutes at 4 °C. To immunoprecipitate endogenous RXR, 400 μg of the lysate was incubated for 1 hour at room temperature with 1 μg of RXR antibody (SC-774, Santa Cruz Biotechnology) complexed with protein A/G magnetic beads. The immune complexes were then washed by IP Lysis/Wash buffer and eluted by Elution buffer. Samples were heated in SDS sample buffer and processed by western blotting with antibodies against HA or VDR.

### Structure models of VDR mutant

The RXR-VDR ligand-binding domain (LBD) heterodimeric model was generated on the basis of the RXR-RAR LBD crystal structure (PDB code: 1DKF)^23^, in which the RAR LBD was replaced by the VDR LBD crystal structure (PDB code: 1DB1) using Discovery Studio (v3.5; Accelrys Inc., San Diego, CA)^41^. The geometry of the model was optimized using the algorithm of smart minimization in a CHARMM force field. The mutant models were constructed using the Built Mutants protocol followed by energy minimization.

## References

[1] Malloy PJ (1990). The molecular basis of hereditary 1,25-dihydroxyvitamin D3 resistant rickets in seven related families. J Clin Invest..

[2] Malloy, P. J., Wang, J., Srivastava, T. & Feldman, D. Hereditary 1,25-digydroxyvitamin D resistant rickets. In Vitamin D (eds Feldman, D, Pike J.M. & Adams, J.) 1197–1232 (Elsevier, 2011).

[3] Malloy PJ, Wang J, Srivastava T, Feldman D (2010). Hereditary 1,25-digydroxyvitamin D resistant rickets with alopecia resulting from a novel missense mutation in the DNA-binding domain of the vitamin D receptor. Mol Genet Metab..

[4] Brown AJ, Dusso A, Slatopolsky E (1999). Vitamin D. Am J Physiol..

[5] Mallory PJ, Pike JW, Feldman D (1999). The vitamin D receptor and the syndrome of hereditary 1,25-dihydroxyvitamin D–resistant rickets. Endocr Rev..

[6] Mallory, P. J., Pike, J. W. & Feldman D. Hereditary 1,25-dihydroxyvitamin D resistant rickets. In Vitamin D (eds Feldman, D, Pike J. M. & Adams, J.) 1207–1238 (Elsevier, 2005).

[7] Malloy PJ (2014). Vitamin D receptor mutations in patients with hereditary 1,25-dihydroxyvitamin D–resistant rickets. Mol Genet Metab..

[8] Nguyen TM (2002). Tryptophan missense mutation in the ligand-binding domain of the vitamin D receptor causes severe resistance to 1,25-dihydroxyvitamin D. J Bone Miner Res..

[9] Malloy PJ, Xu R, Peng L, Clark PA, Feldman D (2002). A novel mutation in helix 12 of the vitamin D receptor impairs coactivator interaction and causes hereditary 1,25-dihydroxyvitamin D–resistant rickets without alopecia. Mol Endocrinol..

[10] Deeb KL, Trump DL, Johnson CS (2007). Vitamin D signalling pathways in cancer: potential for anticancer therapeutics. Nature Rev Cancer..

[11] Whitfield GK (1996). Vitamin D receptors from patients with resistance to 1,25-dihydroxyvitamin D3: point mutations confer reduced transactivation in response to ligand and impaired interaction with the retinoid X receptor heterodimeric partner. Mol Endocrinol..

[12] Macedo LC (2008). Mutations in the vitamin D receptor gene in four patients with hereditary 1,25-dihydroxyvitamin D–resistant rickets. Arq Bras Endocrinol Metab..

[13] Zhou Y, Wang J, Malloy PJ, Dolezel Z, Feldman D (2009). Compound heterozygous mutations in the vitamin D receptor in a patient with hereditary 1,25-dihydroxyvitamin D–resistant rickets with alopecia. J Bone Miner Res..

[14] Rauch F, Lalic L, Glorieux FH, Moffatt P, Roughley P (2014). Targeted sequencing of a pediatric metabolic bone gene panel using a desktop semiconductor next-generation sequencer. Calcif Tissue Int.

[15] Jurutka PW (1997). 1,25-dihydroxyvitamin D3 receptor identifying C-terminal amino acids required acids for transcriptional activation that are functionally dissociated from hormone binding, heterodimeric DNA binding, and interaction with basal transcription factor IIB, in vitro. J Biol Chem..

[16] Liu YY, Nguyen C, Peleg S (2000). Regulation of ligand-induced heterodimerization and coactivator interaction by the activation function-2 domain of the vitamin D receptor. Mol Endocrinol.

[17] Malloy PJ, Zhou Y, Wang J, Hiort O, Feldman D (2011). Hereditary vitamin D-resistant rickets (HVDRR) owing to a heterozygous mutation in the vitamin D receptor. J Bone Miner Res..

[18] Nguyen M (2006). Vitamin D–resistant rickets and type 1 diabetes in a child with compound heterozygous mutations of the vitamin D receptor (L263R and R391S): dissociated responses of the CYP-24 and rel-B promoters to 1,25-dihydroxyvitamin D3. J Bone Miner Res..

[19] Malloy PJ, Zhu W, Zhao XY, Pehling GB, Feldman D (2001). A novel inborn error in the ligand-binding domain of the vitamin D receptor causes hereditary vitamin D–resistant rickets. Mol Genet Metab..

[20] Whitfield GK (1995). A highly conserved region in the hormone-binding domain of the human vitamin D receptor contains residues vital for heterodimerization with retinoid X receptor and for transcriptional activation. Mol Endocrinol..

[21] Jin CH, Kerner SA, Hong MH, Pike JW (1996). Transcriptional activation and dimerization functions in the human vitamin D receptor. Mol Endocrinol..

[22] Nakajima S (1994). The C-terminal region of the vitamin D receptor is essential to form a complex with a receptor auxiliary factor required for high affinity binding to the vitamin D–responsive element. Mol Endocrinol..

[23] Bourguet W (2000). Crystal structure of a heterodimeric complex of RAR and RXR ligand-binding domains. Mol Cell..

[24] Mizwicki MT, Bula CM, Bishop JE, Norman AW (2007). New insights into vitamin D sterol-VDR proteolysis, allostery, structure-function from the perspective of a conformational ensemble model. J Steroid Biochem Mol Biol..

[25] Mazen I, Ismail S, Amr K, Gammal ME, Abdel-Hamid M (2014). Hereditary 1,25-dihydroxyvitamin D-resistant rickets with alopecia in four Egyptian families: report of three novel mutations in the vitamin D receptor gene. J Pediatr ENdocrinol Metab..

[26] Chen CH, Sakai Y, Demay MB (2001). Targeting expression of the human vitamin D receptor to the keratinocytes of vitamin D receptor null mice prevents alopecia. Endocrinology..

[27] Sakai Y, Demay MB (2000). Evaluation of keratinocyte proliferation and differentiation in vitamin D receptor knockout mice. Endocrinology..

[28] Malloy PJ, Feldman D (2011). The role of vitamin D receptor mutations in the development of alopecia. Mol Cell Endocrinol..

[29] Luderer HF, Demay MB (2010). The vitamin D receptor, the skin and stem cells. Mol Biol..

[30] Skorija K (2005). Ligand-independent actions of the vitamin D receptor maintain hair follicle homeostasis. Mol Endocrinol..

[31] Beaudoin GM, Sisk JM, Coulombe PA, Thompson CC (2005). Hairless triggers reactivation of hair growth by promoting Wnt signaling. Proc Natl Acad Sci USA.

[32] Luderer HF, Gori F, Demay MB (2011). Lymphoid enhancer-binding factor-1 (LEF1) interacts with the DNA-binding domain of the vitamin D receptor. J Biol Chem..

[33] Pálmer HG, Anjos-Afonso F, Carmeliet G, Takeda H, Watt FM (2008). The vitamin D receptor is a Wnt effector that controls hair follicle differentiation and specifies tumor type in adult epidermis. PLoS One..

[34] Zou J, Mali P, Huang X, Dowey SN, Cheng L (2011). Site-specific gene correction of a point mutation in human iPS cells derived from an adult patient with sickle cell disease. Blood..

[35] Wallén-Mackenzie Å (2003). Nurr1-RXR heterodimers mediate RXR ligand-induced signaling in neuronal cells. Genes Dev..

[36] Huang K, Malloy P, Feldman D, Pitukcheewanont P (2013). Enteral calcium infusion used successfully as treatment for a patient with hereditary vitamin D resistant rickets (HVDRR) without alopecia: a novel mutation. Gene..

[37] Mechica JB (1997). A novel nonsense mutation in the first zinc finger of the vitamin D receptor causing hereditary 1,25-dihydroxyvitamin D3-resistant rickets. J Clin Endocrinol Metab..

[38] Malloy PJ, Weisman Y, Feldman D (1994). Hereditary 1 alpha,25-dihydroxyvitamin D–resistant rickets resulting from a mutation in the vitamin D receptor deoxyribonucleic acid-binding domain. J Clin Endocrinol Metab..

[39] Huang SM, Cheng YS (2004). Analysis of two CBP (cAMP-response-element-binding protein) interacting sites in GRIP1 (glucocorticoid-receptor-interacting protein), and their importance for the function of GRIP1. Biochemical Journal.

[40] Huang SM, Huang SP, Liu PY (2007). Importin α_1_ is involved in the nuclear localization of Zac1 and the induction of p21^WAF1/CIP1^ by Zac1. Biochemical Journal.

[41] Rochel N, Wurtz JM, Mitschler A, Klaholz B, Moras D (2000). The crystal structure of the nuclear receptor for vitamin D bound to its natural ligand. Mol Cell.

